# A translational approach to studying preterm labour

**DOI:** 10.1186/1471-2393-7-S1-S8

**Published:** 2007-06-01

**Authors:** Rachel Marie Tribe

**Affiliations:** 1 Maternal and Fetal Research Unit, Division of Reproduction and Endocrinology, King's College London, St. Thomas' Hospital Campus, London SE1 7EH, UK

## Abstract

Preterm labour continues to be a major contributor to neonatal and infant morbidity. Recent data from the USA indicate that the number of preterm deliveries (including those associated with preterm labour) has risen in the last 20 years by 30%. This increase is despite considerable efforts to introduce new therapies for the prevention and treatment of preterm labour and highlights the need to assess research in this area from a fresh perspective. In this paper we discuss i) the limitations of our knowledge concerning prediction, prevention and treatment of preterm labour and ii) future multidisciplinary strategies for improving our approach.

## Background

Preterm labour continues to be a major contributor to neonatal and infant morbidity. Recent data from the USA indicate that the number of preterm deliveries (including those associated with preterm labour) has risen in the last 20 years by 30% (from 9.4% to 12.5%) [1,2], with the highest rates still affecting ethnic and racial minorities and women of low socio-economic status. This increase, despite considerable efforts to introduce new therapies for the prevention and treatment of preterm labour, highlights the need to assess research in this area from a fresh perspective.

**Figure 1:**
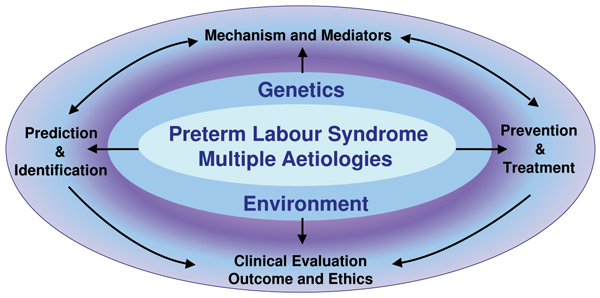
Preterm labour is a complex syndrome of multiple aetiologies requiring a multidisciplinary research approach.

Part of the problem is that preterm labour is a syndrome of multiple aetiologies influenced by a wide number of genetic, biological/biophysical, psychosocial and environmental factors (Figure 1). The emerging consensus is that future research efforts need to focus on improving our ability to predict and diagnose preterm labour by increasing our understanding of the pathophysiological mechanisms involved. A greater mechanistic understanding of preterm labour, coupled with better tools for prediction, would undoubtedly contribute to the appropriate stratification of women into risk groups and facilitate the development of targeted therapeutic agents and timing of clinical intervention. Such studies, however, require a multidisciplinary outlook and a co-ordinated approach to planning and funding research programmes.

### Mediators and mechanisms

For reasons described above, it is highly unlikely that there is one underlying mechanism that will explain preterm labour in all women. However, two contributory processes of current interest include the influence of infection and inflammation, and uterine stretch on myometrial contractility [for details see [3]]. Infection or sub clinical infection is thought to occur in at least 40% of preterm deliveries and more frequently with births that occur below 26 weeks [4] and uterine stretch is proposed to influence uterine contractility to a greater degree in multiple pregnancy. Our understanding of these, and other processes associated with preterm labour, is still relatively limited, although microarray and proteomic studies of uterine tissues and fluids should provide further insight and novel research directions. However, subsequent determination of the functional impact of any mRNA and protein changes will be fundamental to future progress. Similarly, the complex interplay of these processes in different uterine tissues (e.g. cervix, fetal membranes, deciduas and myometrium), as well as the influence of maternal/fetal genetics and environment, will also require in-depth investigation.

### Prediction and identification of women at risk of preterm labour

A large number of studies have attempted to identify a single, or combination of biomarkers (e.g. cytokines, fetal fibronectin etc.) and physical measurements (cervical length) for the detection of women at risk of preterm labour (Table 1) [4-6]. Of interest is the number of inflammation related biomarkers that have been identified. However, the limitation of such data, with the exception a few studies of fetal fibronectin and bacterial vaginosis studies, is that often measurements have been obtained from a single clinical visit at 20–24 weeks' gestation, which at best can provide a snap shot of dynamic processes. Also, the timing of measurements is not necessarily optimal for the detection (and treatment) of women destined to go into early preterm labour (< 26 weeks'). Of the myriad markers identified, only cervical length and bedside fetal fibronectin measurement appear to be adopted into research based clinical practice in the UK. As a result, there remains a reliance on previous pregnancy history for the identification of high-risk women within current antenatal care, which precludes the identification of women in their first pregnancy.

Advances in '-omic' technology (proteomics, metabolomics, peptidomics) may offer an alternative strategy for detection of women at risk of preterm labour regardless of parity [7,8]. Indeed, several informative peptides have been reported, including calgranulins, and defensins, using this approach [9-12]. Currently, these investigations tend to be limited to analysis of single biological samples from women either in established preterm labour or known to be of increased risk. Future studies would benefit from collecting serial samples from women in order to gain mechanistic insight into disease progression, as well as identifying candidate biomarkers for testing in low risk populations.

### Tocolytic and prophylatic treatment of women

Despite an incomplete understanding of mechanisms underlying preterm labour, a plethora of different intervention strategies have been attempted. Administration of tocolytics in symptomatic women achieves at best only one or two days extension of gestation, and none of the classes of drugs in clinical use is clearly more effective than any other [13]. Results from recent trials of prophylactic therapy (e.g. prostaglandin endoperoxide synthase 2 (PTGS2) inhibitors and antibiotics) [14-18], with the exception of vaginal progesterone [19,20], have been disappointing. This may be, in part, due to our inability to identify those women most likely to benefit from treatment and a lack of consensus as to when to intervene, a situation that would be greatly improved by more knowledge of the pathophysiological events involved.

### Current translational research strategy

**Figure 2:**
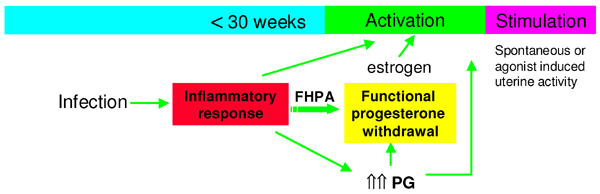
**Schematic diagram to illustrate potential mechanisms by which infection/inflammation could influence uterine smooth muscle culminating in premature labour**. Aspects of this model are also relevant to the processes influencing the cervix and fetal membranes. FHPA, fetal hypothalamic pituitary adrenal axis; PG, prostaglandins.

Our approach to preterm labour research attempts to address some of the criticisms raised above. With support of Tommy's the baby charity, we have created a multidisciplinary environment for pregnancy research which encourages bidirectional interactions between scientists and clinicians. Our current strategy has resulted in the development of common research themes and collaborative translational studies to address scientific and clinical questions concurrently. One such theme is the understanding the role that inflammatory mediators play in preterm labour (Figure 2).

**Figure 3:**
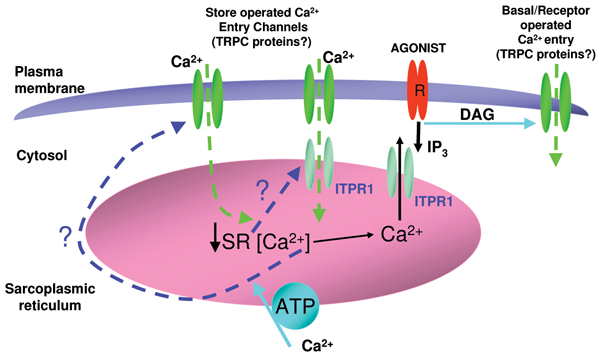
**Calcium entry pathways associated with TRPC proteins**. TRPC proteins are proposed to form store operated and basal/receptor operated plasma membrane calcium channels (depicted in green). Receptor operated calcium entry can occur when an agonist (e.g. oxytocin or PGF_2α_) binds to its receptor (R) resulting in an increase in diacylglycerol (DAG) and IP_3 _(inositol 1,4,5-trisphosphate). Interaction of IP_3 _with the receptor (ITPR) causes release of calcium for the sarcoplasmic reticulum (SR) and initiates store operated calcium entry.

Basic research studies have concentrated on the mechanisms by which the pro-inflammatory cytokine interleukin (IL) 1B might influence uterine smooth muscle excitability. It is well documented that inflammatory cytokines stimulates PTGS2 expression and prostaglandin synthesis in human myometrium [21,22]. However, in light of the disappointing clinical results studies using PTGS2 inhibitors [14], our focus has been on the alternative pathways, particularly transient receptor potential cation channel subfamily C proteins (TRPC1-7; that can form calcium entry channels), by which cytokines may influence uterine contractility (Figure 3). The TRPC gene family encode for proteins that form cation entry channels associated with store operated calcium entry (also known as capacitative calcium entry) and receptor/basal calcium entry channels [23]. The activation of such channels is responsible for mediating a wide range of physiological processes including augmentation of agonist-induced contraction, cell growth and calcium sensitive induced changes in gene expression [23]. TRPC proteins have been identified in both lower and upper segment tissue and cells [24-27]. We have demonstrated a significant increase in TRPC3, 4 and 6 protein in myometrial tissue from women in labour and have subsequently investigated potential stimuli responsible for inducing such changes [26]. The working hypothesis proposes that cytokines will i) induce calcium signals that regulate gene expression of cellular proteins, including components of the prostaglandin signalling cascade, that promote uterine contraction and ii) specifically enhance expression of pathways involved in increasing calcium availability for contraction.

Our recent studies have clearly demonstrated that IL1B, at concentration found in high vaginal swabs from women in preterm labour, increases the excitability of human uterine cells [28] within 24 hours of treatment. This timing is consistent with *in vivo *observations that IL1B induces contractions in animals within a similar timeframe [29]. Specifically, IL1B modulates protein expression of TRPC3 (a putative calcium channel) and SR Ca^2+^-ATPases, initiates spontaneous [Ca^2+^]_i _oscillations and augments calcium influx pathways and a store operated current [26,28]. Interestingly, the IL1B induced increase in TRPC3 expression does not appear to be influenced by PTGS2 mediated prostaglandin synthesis, as nimesulide (a PTGS2 specific inhibitor) does not inhibit the response [26]. However this does not rule out the possibility that prostaglandins, arachidonic acid or components of the prostaglandin-receptor mediated response stimulates calcium entry via TRPC3 channels. The induction of TRPC3 protein expression by IL1B may in part explain the increase in TRPC3 expression observed in uterine muscle at labour onset and provides a plausible mechanism by which cytokines could induce premature labour. This work is being expanded upon in order to further dissect the signalling cascade and determine whether inflammatory agents enhance calcium signalling pathways and modulate human myometrial tissue function.

To complement our scientific research, we have instigated a new study (CLIC: a mechanistic investigation into cervical length and inflammatory changes; funded by Action Medical Research). It is recognised that preterm labour is often pre-empted by progressive asymptomatic cervical shortening; inflammatory mediators (e.g. IL6, IL1B and tumour necrosis factor) and matrix metalloproteinases are implicated in this premature ripening of the cervix [30]. However, it remains unclear whether inflammatory responses precede cervical shortening or are a consequence of labour itself. This mechanistic longitudinal study will address the question as to whether inflammation is associated with cervical shortening and if commonly used intervention impact on the process. Women are being recruited from several high-risk preterm clinics (in compliance with the Declaration of Helsinki, 1996) [31] and a range of longitudinal biochemical and biophysical measurements recorded. A variety of 'omic' based technologies will also be employed to provide insight into the temporal nature of biochemical events and potentially identify novel biomarkers for prediction of risk.

## Conclusion

If we are to make any impact in the area of prevention, prediction and treatment of preterm labour we need to revisit our current strategies and develop a more multidisciplinary approach to undertaking research in this area. The experience of our collaborative grouping suggests that close interactions between scientists and clinicians enables the development of common research themes targeted at providing insight into one aspect of the complex aetiology of preterm labour. It is anticipated that the development of similar, but larger research networks will ultimately be the key to understanding the complete syndrome of preterm labour and the improvement of outcomes for mothers and babies.

## Competing interests

The author declares that they have no competing interests.

## Figures and Tables

**Table 1:** Examples of the wide range of biochemical and biophysical markers associated with potential risk of preterm labour

**Potential predictors of preterm birth**
Bacterial vaginosis
Interleukin 6
Interleukin 1B
Interleukin 8
Tumor necrosis factor (TNF superfamily, member 2)
Colony stimulating factor 3
Relaxin
C-terminal propeptide of procollagen
Fetal fibronectin
*α*-fetoprotein
Salivary estriol
Cervical length
Electromyograph activity
Maternal body mass index
Previous history
Human chorionic gonadotrophin
Corticotrophin releasing hormone

For further details see [4-6].
